# Supplementary figures and images for: Highly-potent, synthetic APOBEC3s restrict HIV-1 through deamination-independent mechanisms
Source: PLoS Pathog. 2021 Jun 25;17(6):e1009523. doi: 10.1371/journal.ppat.1009523 (PMC8266076; doi:10.1371/journal.ppat.1009523)

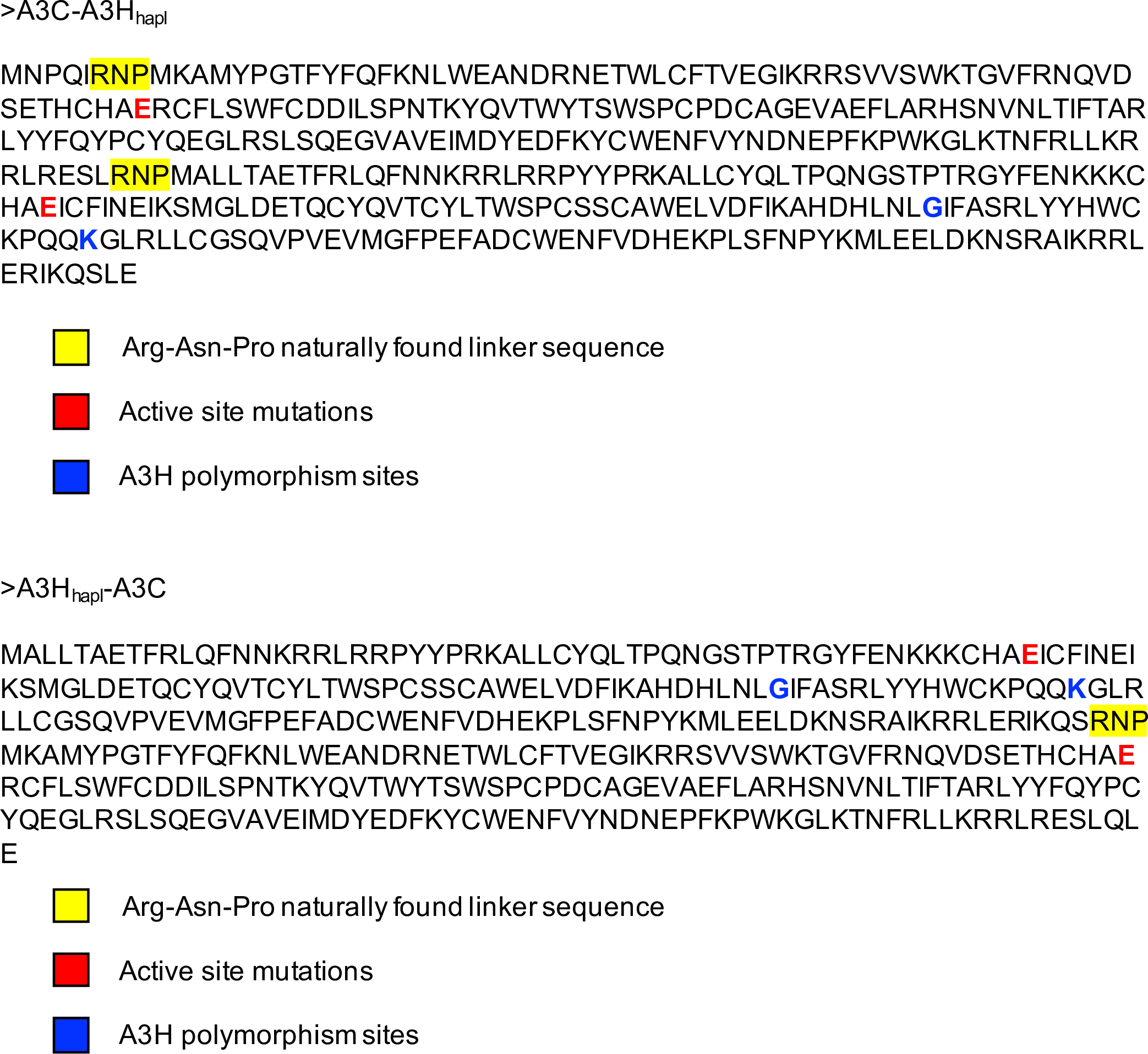

Supplement: S1 Fig — Full length sequences used to construct the A3C-A3H and A3H-A3C double deaminase domains. The “RNP” amino acid sequence (Arginine-Asparagine-Proline) that links the two deaminase domains together is highlighted in yellow. The essential glutamic acid that is necessary for deaminase activity is in red text. The A3H human polymorphic sites are shown in blue text. (TIF) [file ppat.1009523.s001.tif]

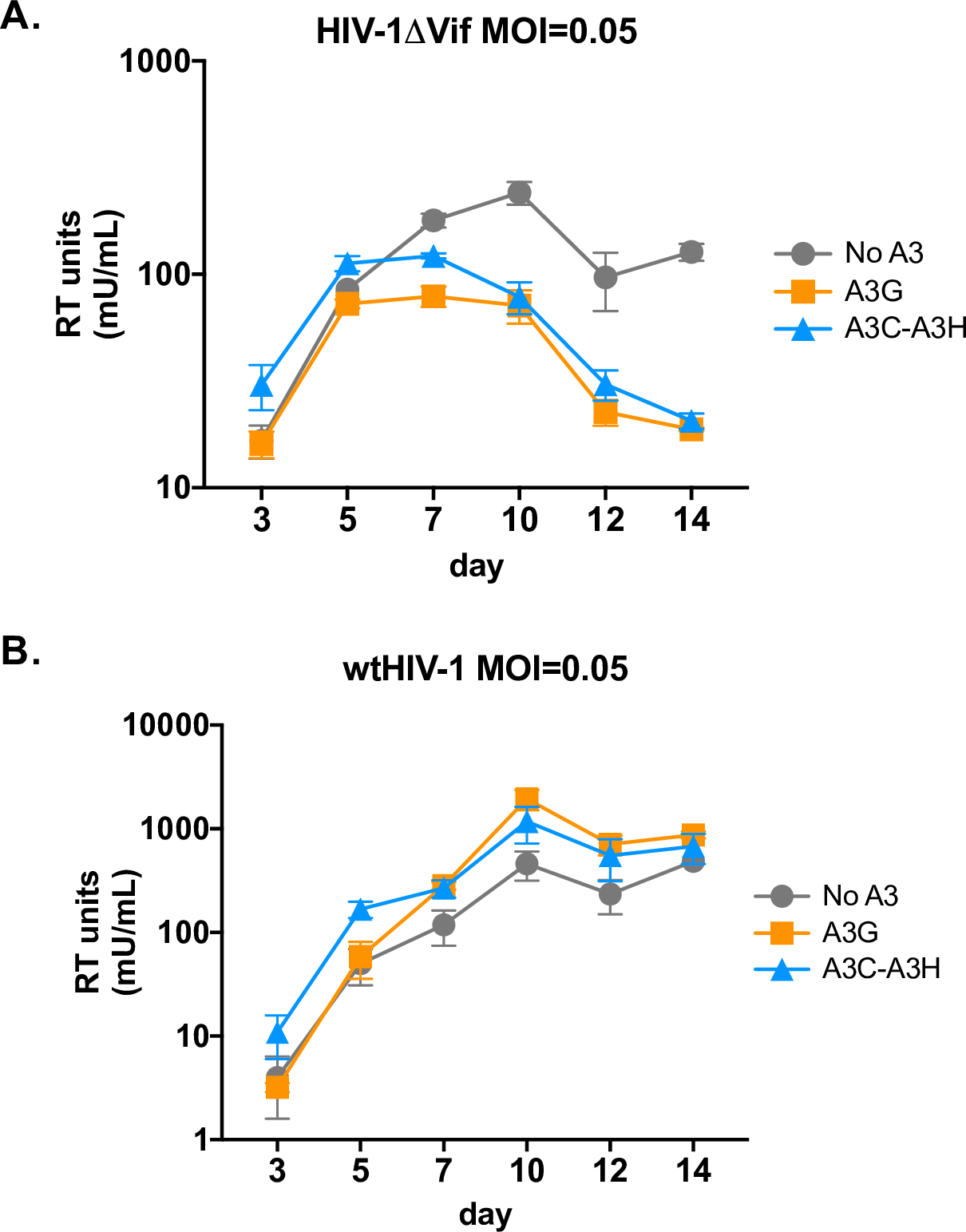

Supplement: S2 Fig — Spreading infection kinetics of a replication-competent HIV-1 with a deletion that spans the Vif open reading frame (called HIV-1ΔVif) (A) or wtHIV-1 (LAI isolate) (B). The Jurkat cells expressing no A3 (circle, grey line), A3G (square, orange line), or A3C-A3Hhap II (celled A3C-A3H, triangle, blue line) were infected at a low MOI (MOI = 0.05) in triplicates. Virus production was monitored over time by collecting supernatant and measuring RT activity (mU/mL) using a SG-PERT assay. Error bars represent the standard error across the 3 biological replicates. (TIF) [file ppat.1009523.s002.tif]
