# Supplementary material for: Highly-potent, synthetic APOBEC3s restrict HIV-1 through deamination-independent mechanisms
Source: PLoS Pathog. 2021 Jun 25;17(6):e1009523. doi: 10.1371/journal.ppat.1009523 (PMC8266076; doi:10.1371/journal.ppat.1009523)
Supplement: Attachment — Submitted filename: A3C-A3H paper responses.docx [file ppat.1009523.s003.docx]

Reviewer #1: In this manuscript, the authors explore combining deaminase domains from individual APOBEC proteins to determine whether this would increase antiviral activity against HIV. The double deaminase domain protein APOBEC3G is the most potent of the APOBECs in restricting HIV, and it is currently unknown whether all of the possible deaminase domain combinations have been evolutionarily explored in nature. The authors determine that combining deaminase domains from APOBEC3C and 3H results in a super-restrictor that limits HIV replication at least as well as or better than APOBEB3G depending on which assay they utilize. They mechanistically determine that the strong antiviral activity is a result of both deaminase activities and enhanced interaction of the novel restriction factor with viral RNA, correlating with increased packaging into virions. Though the practical/translational impacts of the work are unclear, the experiments are very well done, the data are presented clearly, and the results are informative with regard to the evolutionary history and the breadth of evolutionary sampling of possible antiviral APOBECs.

Reviewer #2: In this work the authors demonstrated that a synthetic A3C/A3H di-domain chimera acquired more efficiency in virion packaging and enhanced antiviral activity, which is comparable to the most potent natural di-domain A3G, and the antiviral activity is conferred by both deaminase-dependent and independent mechanisms. The authors also showed that the deaminase-independent activity is correlated to the increased RNA binding affinity of the A3C/A3H chimera. This is a clean work with only minor modifications needed.

Response: We thank the reviewers for their kind words.

1. Figure 4 only shows the anisotropy measurements for A3C-A3H, A3H and A3G. How about the others, e.g., A3H-A3C and A3C?

Response: We would have liked to do A3H-A3C as well. However, this protein produced very poorly in insect cells and we were not able to obtain enough protein to do the assay properly. Because of that, we focused Figure 5 on the A3C-A3H super restriction factor so that we could match biochemistry with the effects of this protein in HIV spreading infections. We did not test A3C since previous results showed that A3C binds RNA much less strongly than A3H (we now cite those references in the main text), therefore we considered A3H to be the more relevant comparison. We have added this rationale on Page 13, line 274.

2. In Figure 5C the No-A3 control has considerable level of G-to-A mutations. Why is it so different from that in Figure 3A?

Response: We agree that we should have explained this better. Figure 3A is a single round infection, while the mutation assay in Figure 5C is after 14 days of a spreading infection in T cells. Therefore, one would expect more G to A mutations due to reverse transcriptase errors during replication (it is known that RT has a G to A bias) and possibly a low level of A3 expression in Jurkat T cells. We now more explicitly point out how the differences between the assays will affect these results and where the source of the G to A mutations might come from on Page 15, line 328.

**Part II – Major Issues: Key Experiments Required for Acceptance**
Please use this section to detail the key new experiments or modifications of existing experiments that should be absolutely required to validate study conclusions.

Generally, there should be no more than 3 such required experiments or major modifications for a "Major Revision" recommendation. If more than 3 experiments are necessary to validate the study conclusions, then you are encouraged to recommend "Reject".

Reviewer #1: Figure 2 is representative of 3 experiments. Can the results from the 3 experiments be quantified and included as a bar graph?

Response: This is a good idea. We have now added this data as Figure 2, panel B.

The usefulness of the research is not entirely clear. The authors have coined the term “super-restrictor,” and their novel proteins could potentially be useful in future gene therapy approaches to HIV treatment. However, all of the assays are done with HIV-deltaVIF, which is sensitive to APOBEC inhibition. Creating an APOBEC-based super-restrictor that evades VIF inhibition and can thus inhibit WT HIV-1, would be a more exciting advance. Based on published literature regarding the VIF-APOBEC interaction interferface, can the authors create and test such a version of the A3H/A3C constructs?

Response: We consider the usefulness to be more basic rather than as a gene therapy approach. That is, we addressed the question about the evolutionary potency of the *A3* locus in general. We do agree with the reviewer that it might have been more exciting if these constructs were resistant to Vif (although we still think it is very instructive that they are sensitive to Vif). We have started a few preliminary experiments to ask if mutations at predicted interfaces on either the A3H, the A3C, or both sides of the super restriction factor will render it Vif-resistant. However, we feel that this will be a long term project and would prefer not to wait for those results in the present manuscript.

Reviewer #2: (No Response)
